# Synthesis, experimental and computational studies on the anti-corrosion performance of substituted Schiff bases of 2-methoxybenzaldehyde for mild steel in HCl medium

**DOI:** 10.1038/s41598-023-30396-3

**Published:** 2023-02-24

**Authors:** Collins U. Ibeji, Damilola C. Akintayo, Henry O. Oluwasola, Eric O. Akintemi, Onyinye G. Onwukwe, Onyeka M. Eziomume

**Affiliations:** 1 Department of Pure and Industrial Chemistry, Faculty of Physical Sciences, University of Nigeria, Nsukka, 410001 Enugu State Nigeria; 2 Catalysis and Peptide Research Unit, School of Health Sciences, University of KwaZulu-Natal, Westville Campus, Durban, 4041 South Africa; 3 School of Chemistry and Physics, University of KwaZulu-Natal, P.M.B. X54001, Durban, 4000 South Africa; 4 Industrial Chemistry Unit, Department of Computer and Physical Sciences, Wesley University, P. M. B. 507, Ondo, Nigeria; 5 Faculty of Chemistry, University of Warsaw, 02-093 Warsaw, Poland

**Keywords:** Computational models, Chemistry

## Abstract

Corrosion inhibition performance of two synthesized Schiff base ligands; (E)-2-((2-methoxybenzylidene)amino)phenol **L1** and (E)-2-((4-methoxybenzylidene)amino)phenol **L2** were carried out by weight loss measurement in 0.1 M hydrochloric acid (HCl) solution. Density Functional Theory (DFT) and Molecular dynamics (MD) simulation were applied to theoretically explain the inhibitors’ intrinsic properties and adsorption mechanism in the corrosion study. The result of the inhibition performances carried out at varying concentrations and temperatures were compared. The corrosion inhibition efficiencies of **L1** and **L2** at an optimal concentration of 10 × 10^–4^ M were 75% and 76%. Langmuir isotherm model fits the data obtained from the experiment with a correlation coefficient (R^2^) value closer to unity. The adsorption mechanism of inhibitor on the surface of the Fe metal occurred via chemisorption inferred from the Gibbs free energy (ΔG_ads_). Scanning electron microscopy showed a mild degradation on the surface of the mild steel immersed in the **L1,** and **L2** inhibited acid solution, which could be due to surface coverage. The energy dispersive X-ray spectroscopy showed the metal surface’s elemental composition and the existence of the chlorine peak, which emanates from the HCl medium. DFT calculations revealed that the hybrid B3LYP functional performed better than the M06-2X meta-functional in estimating the energies of the synthesized Schiff bases for corrosion inhibition as seen in the lower ΔE values of 3.86 eV and 3.81 eV for **L1** and **L2.** The MD simulation revealed that the orientation of inhibitors on the surface of the metal resulted in the coordination bond formation and that the interaction energy of **L2** was −746.84 kJ/mol compared to −743.74 kJ/mol of **L1.** The DFT and MD results agreed with the observed trend of the experimental findings.

## Introduction

Iron-based materials and low-alloyed metals are useful in several industrial applications and, they play important roles due to their structural and mechanical strength^1–3^. Mild steel is a type of ferrous alloy that has been used extensively in various industrial applications such as in the fabrication of cooling systems, pipes, and space vehicles^4,5^. These metals and alloys become corroded due to the application of mineral acids^6–8^. Corrosion inhibition of mild steel using organic compounds, including natural products^9,10^, synthetic and some inorganic compounds^7,11^ has attracted much interest in recent years. These compounds serve as corrosion inhibitors because of the presence of heteroatoms such as nitrogen, oxygen, sulphur and π-electrons that promote adsorption on the metal surfaces thereby minimizing the deterioration of metals and their alloys in an acid medium ^2^. Schiff bases have been reported to be excellent corrosion inhibitors of corrosion due to the presence of the imine group that coordinates with the metal ions^3^. The inhibition efficiency of any given Schiff base ligand depends primarily on the type of *P*-orbitals, orbitals of the metal ions, and the acid medium^12,13^. The structural and electronic properties of compounds are important in the corrosion inhibition mechanism. Therefore, to comprehend the mechanism of inhibition between the surface of the metal and the inhibitor, Monte Carlo Simulation, molecular dynamics and quantum mechanics calculations are expedient^14^. Many accounts regarding Schiff bases as corrosion inhibitors have been reported, however, scanty work exists on the corrosion inhibition effect of ortho and para-directing groups in compounds as corrosion inhibitors. Therefore, this work reports the synthesis, and corrosion inhibition effects of ortho and para-substituted (E)-2-(methoxybenzylidene)amino)phenol Schiff base ligands on mild steel in 0.1 M HCl. In this study, the gravimetric (weight loss) method was adopted and scanning electron microscopy (SEM) and energy dispersive X-ray (EDX) spectroscopy were applied to analyse the metal surface. Density Functional Theory (DFT), Monte Carlo and molecular dynamics Simulation was used to understand the adsorptive capacity and mechanism inhibitor and metal ion interactions.

## Experimental methods

### Materials and methods

2-aminophenol, 2-methoxybenzaldehyde, and 4-methoxybenzaldehyde were purchased from Sigma-Aldrich and used without further purification. The ^1^H- and ^13^C-NMR spectra were acquired in CDCl_3_ at room temperature using a Bruker 400 MHz spectrometer, and their data were recorded in CDCl_3_, with a residual internal solvent signal of 7.26 and 77.00 ppm, respectively. On a Perkin Elmer FTIR spectrometer equipped with universal ATR, FT-IR spectra reported in wavenumbers (cm^−1^) were obtained. Mass spectra of the compounds were obtained from a Water synaptic GR electrospray positive spectrometer. The electronic absorption spectra were obtained using a UV-3600 Shimadzu UV–VIS-NIR spectrophotometer. Thermo Scientific FLASH 20 0 0 CHNS/O Analyzers were used for Elemental analyses.

### General synthesis of the Schiff base ligands

**Figure 1:**
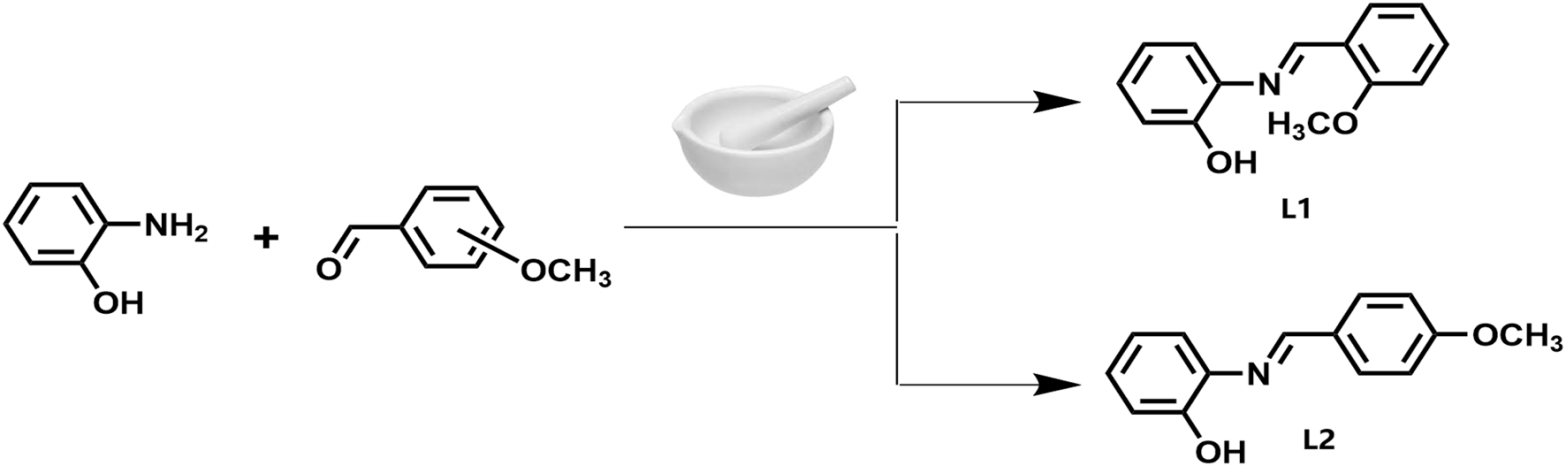
Synthetic procedure for Schiff base ligands.

The Schiff base ligands (E)-2-((2-methoxybenzylidene)amino)phenol **L1** and (E)-2-((4-methoxybenzylidene)amino)phenol **L2** were obtained in excellent yields (97–98%) by solvent-free grinding using a mortar and pestle (Fig. 1) according to commonly described protocol in literature ^15,16^. The resultant products were dried in *vacuo* to completely remove the water and characterized spectroscopically using IR, NMR, and UV–Vis.

### Synthesis of L1

2-Aminophenol (1 mmol, 0.109 g) was ground with 2-methoxybenzaldehyde (1 mmol, 0.136 g) for 10–12 min to obtain (E)-2-((2-methoxybenzylidene)amino)phenol **L1** as a yellow solid. Yield (3.36 g, 97%), M.Pt.: 100–102 °C. ^1^H-NMR (CDCl_3_ 400 MHz) δ (ppm) 3.95 (s, 3H), 6.92 (t, *J* = 7.66 Hz, 1H), 7.01 (dd, *J* = 8.24, 12.37 Hz, 2H), 7.08 (t, *J* = 7.56 Hz, 1H), 7.20 (t, *J* = 7.84 Hz, 1H), 7.35 (d, *J* = 7.92 Hz, 1H), 7.49 (t, *J* = 7.82 Hz, 1H), 8.18 (d, *J* = 7.72 Hz, 1H), 9.21 (s, 1H). ^13^C-NMR (CDCl_3_ 400 MHz) δ (ppm) 153.1 (CH), 133.0 (C), 128.5 (C), 127.4 (C). 120.09 (CH), 120.0 (CH), 116.1 (CH), 114.8 (CH), 111.3 (CH), 55.6 (CH_3_). FT-IR (KBr, cm^−1^): 3329 ѵ (O–H), 2933 ѵ (sp^3^ C–H), 1587 ѵ (CH=*N*). MS-TOF m/z (%): 284.06 [*M* + H]^+^. Anal. Calcd. for C_14_H_13_NO_2_: C, 73.99; H 5.77; N 6.16%. Found; C, 73.80; H 5.59; N 6.06.

### Synthesis of L2

2-Aminophenol (1 mmol, 0.109 g) was mixed with 2-methoxybenzaldehyde (1 mmol, 0.136 g) to obtain (E)-2-((4-methoxybenzylidene)amino)phenol **L2** as a yellow solid. Yield (3.36 g, 98%), M.pt.: 83–85 °C. ^1^H-NMR (CDCl_3_ 400 MHz) δ (ppm) 3.91 (s, 3H), 6.92 (t, *J* = 7.64 Hz, 1H), 7.02 (d, *J* = 8.68 Hz, 2H), 7.03 (d, *J* = 6.28 Hz, 1H), 7.22 (t, 1H), 7.29 (d, 1H), 7.49 (t, *J* = 7.82 Hz, 1H), 7.90 (d, *J* = 8.71 Hz, 2H), 8.65 (s, 1H). ^13^C-NMR (CDCl_3_ 400 MHz) δ (ppm) 162.5 (CH), 156.6 (C), 152.1 (C), 135.9 (C), 130.6 (C), 128.9 (CH), 128.3 (CH), 120.1 (CH), 115.8 (CH), 114.8 (CH), 114.3 (CH), 55.5 (CH_3_). FT-IR (KBr, cm^−1^): 3338 ѵ (O–H), 2836 ѵ (sp^3^ C –H), 1589 ѵ (CH=*N*). MS-TOF m/z (%): 284.06 [*M* + *H*]^+^. Anal. Calcd. for C_14_H_13_NO_2_: C, 73.99; H 5.77; N 6.16%. Found; C, 73.85; H 5.55; N 6.10.

### Metal and tests solution preparation

The carbon steel used in this corrosion study was procured commercially and cut to 4.0 cm × 3.0 cm × 0.7 cm in dimensions. The sample weight (%) composition is 0.17% C, 0.26% Si, 0.46% Mn, 0.017% S, 0.019% P, and the remaining Fe. The samples were abraded with emery papers of varying grades (grade 80–2000), degreased with ethanol and acetone to clean the surface of dust particles and stains, then rinsed with distilled water and further dried at room temperature and stored in moisture-free desiccators prior to the weight loss experiment. The test solution of 0.1 M HCl was prepared with distilled water by adequate dilution of analytical grade 37% HCl (from MERCK) with distilled water.

### Weight loss measurement

The prepared mild steel samples were weighed accurately in triplicate with a sensitivity of ± 0.1 mg then immersed in the corrosive solution of (100 mL, 0.1 M HCl) blank and at different concentrations of the Schiff base inhibitors kept at 303 K, 333 K and 363 K respectively in a regulated thermostat water bath for 3 h. Afterwards, the samples were removed from the acidic solution, thoroughly washed with distilled water, dried in a vacuum oven, and then re-weighed. Analysis carried out was in triplicate after which the mean value of the weight loss measurements was reported. The average weight loss (ΔW), corrosion rate (CR), degree of surface coverage (θ) and inhibition efficiency ($\eta {\text{w}}$) were calculated using Eqs. 1–4, respectively.

1 $$\Delta \mathrm{W }(\mathrm{g})={\mathrm{W}}_{\mathrm{bimm}}-{\mathrm{W}}_{\mathrm{aimm}}$$

2 $$\mathrm{CR }(\mathrm{mg}/{cm}^{2}\mathrm{h}) = \frac{{\mathrm{W}}_{\mathrm{bimm}}-{\mathrm{W}}_{\mathrm{aimm }}}{\mathrm{A x t}}$$

3 $$\theta = \frac{C{R}_{blank }- C{R}_{inh}}{C{R}_{blank}}$$

4 $$\eta {\text{w }}\left( \% \right) = \frac{{CR_{blank } - CR_{inhh} }}{{CR_{blank} }} \times 100$$

$\Delta \mathrm{W}$ is the Weight loss (g) of the mild steel coupons, ${\mathrm{W}}_{\mathrm{bimm }}\mathrm{and} {\mathrm{W}}_{\mathrm{aimm}}$ are the weights (g) of mild steel before immersion and after immersion; A is the area of the mild steel coupons (cm^2^), and t is the exposure time (h); CR_blank_ is corrosion rates without the inhibitor and CR_inh_ is corrosion rates in the presence of inhibitor.

### Surface analysis

The prepared mild steel samples were immersed in a test solution of 0.1 M HCl (100 mL) with and without 1 mM concentration of **L1** and **L2** inhibitors in their respective beakers at 303 K and were subjected to an exposure period of 3 h in a thermostat water bath and afterwards removed from the solution and rinsed using distilled water and acetone respectively. It was allowed to dry and stored in a desiccator before the analysis of the surface morphology. The scanning electron microscope on (SEM, Quanta 450 FEI, voltage 10 kv, spot size 9 mm and magnification range 2228–2382 with 10 μm scale bar model: Σigma HD, Zeiss, Germany).

### Quantum mechanics calculations

The DFT quantum mechanics calculations were performed to evaluate the molecule’s chemical properties, including atomic charge and intra-molecular geometry ^17^; stereo-electronic interactions accountable for conformational stability ^18^, and the selection of corrosion inhibitors ^19^ among others. In this study, the popular density functional theory (DFT)^20^ method embedded in Gaussian 16 program ^21,22^ was used to investigate the electronic properties of newly synthesized Schiff bases for their reactivity and mechanism as a corrosion inhibitor. The hybrid density functional B3LYP ^23^ and the highly parameterized exchange-correlated M06-2X ^24^ functionals in combination with the 6-31 + G(d,p) basis set ^23^ were chosen for the calculations and the results compared for their performances with respect to each variable. The Schiff bases **L1** and **L2** were modelled with GaussView 6 program and optimized to a minimum in the gas-phase. The frequency calculations were performed on optimized geometries both in gas-phase and hydrochloric, HCl (ε = 78.3, experimental solvent) media; and the results were compared for solvation effect. The fundamental conceptual DFT properties measured are the energies of the lowest unoccupied molecular orbital, E_LUMO_ and the highest occupied molecular orbital, E_HOMO_. Other conceptual DFT parameters were determined from these basic properties in line with the work of Odewole et al. ^3^ as expressed in Eqs. (5) to (12).

5 $$\Delta E= {E}_{LUMO}- {E}_{HOMO}$$

6 $$A= -{E}_{HOMO}$$

7 $$I= {-E}_{LUMO}$$

8 $$\eta = \frac{\Delta E}{2}$$

9 $$\delta = \frac{1}{\eta }$$

10 $$\chi = \frac{(I+A)}{2}$$

11 $${C}_{P}= -\chi$$

12 $$\omega = \frac{{\chi }^{2}}{\Delta E}$$

where ΔE is the energy gap, A is the electron affinity, I is the ionization potential, η is the chemical hardness, δ is the softness, χ is the absolute electronegativity, C_P_ is the chemical potential, and ω is the global electrophilicity index.

The mechanism of inhibition was measured in terms of how electrons were transferred vis-à-vis the inhibitor and metal surface. In this case, the fraction of electron transferred, (ΔN) onto the metal surface was determined from the electronegativity and chemical hardness of iron (Fe) and the inhibitor as expressed in Eq. (13).

13 $$\Delta N= \frac{{\chi }_{Fe}- {\chi }_{inh}}{2({\eta }_{Fe}- {\eta }_{inh})}$$

χ_Fe_ and χ_inh_ are the absolute electronegativities of iron and the inhibitor respectively while η_Fe_ and η_inh_ are the chemical hardness of iron and inhibitor respectively. The theoretical values 7 for χ_Fe_ and 0 for η_Fe_ were used according to a previous study by Ebenso and co-workers ^25^.

The local reactivity at various sites of the ligands was treated from the Mulliken atomic charges. These charges form the basic variables for deriving Fukui functions, namely nucleophilicity and electrophilicity, of the molecules according to Eqs. (14) to (16) and were used to examine the potential adsorption sites on the Schiff base molecules.

14 $${f}_{k}^{+}= \left[{q}_{k}\left(N+1\right)- {q}_{k}\left(N\right)\right]$$

15 $${f}_{k}^{-}= \left[{q}_{k}\left(N\right)- {q}_{k}\left(N-1\right)\right]$$

16 $$\Delta {f}_{k}\left(r\right)= {f}_{k}^{+}- {f}_{k}^{-}$$

where f_k_^+^ and f_k_^−^ are the nucleophilic and electrophilic Fukui functions, respectively. q_k_(N + 1) and q_k_(N − 1) are the charges on the atoms in a molecule in its anionic and cationic states, respectively. q_k_(N) is the charge on the atoms in a molecule in its neutral state.

### Molecular dynamics simulation

Molecular dynamics (MD) simulation is known and has been applied to investigate the interaction of metal (Fe) surfaces and inhibitors. The MD simulation was carried out using Material Studio (Accelrys Inc)^26^. For better stability and the nature of the packed surface of the chosen metal surface, Fe (1 1 0) was used in this study. Fe (1 1 0) surface also is one of the most active to stimulate the adsorption process. The relations between the inhibitor and the Fe (1 1 0) surface were performed in a simulation box size of 24.82 × 24.82 × 26.14 Å^3^ thru periodic boundary conditions. A vacuum slab of 6 Å height was set up on the Fe (1 1 0). 200 molecules of water, 9 of Cl^−^ and 9 of H_3_O^+^ were included using the Adsorption locator module in Material Studio ^27^. Geometric optimization was performed to obtain minimal structures. 5 cycles (2000 steps) of simulated annealing were applied and the MD was performed via Berendsen thermostat temperature control at 298 K and simulation time of 50 ps with 1 fs time step^28^. The simulation was carried out under canonical ensemble (NVT) and Condensed Phase Optimized Molecular Potentials for Atomistic Simulation Studies (COMPASS) force field^2,28,29^. The interaction energy was obtained, and the Radial Distribution Function (RDF) was computed. The interaction energies were obtained based on Eq. 17.^28^

17 $${\mathrm{E}}_{\mathrm{interaction }= }{\mathrm{E}}_{\mathrm{total}}-({\mathrm{E}}_{\mathrm{surface}+ {\mathrm{H}}_{2}\mathrm{O}+{\mathrm{H}}_{3}{\mathrm{O}}^{+}+{\mathrm{Cl}}^{-}+}{\mathrm{E}}_{\mathrm{inhibitor}})$$

E_total_ represents the total energy of the inhibitor molecule, surface, H_2_O, H_3_O and Cl^−^, *E*_inhibitor_ designates the energy of absorbed inhibitor on the surface and the *E*_surface_ implies the energy of the metal surface involving H_2_O, H_3_O and Cl^−^.

18 $${\mathrm{E}}_{\mathrm{binding }}{=-\mathrm{E}}_{\mathrm{interaction}}$$

## Results and discussion

### Spectroscopic studies

**Figure 2:**
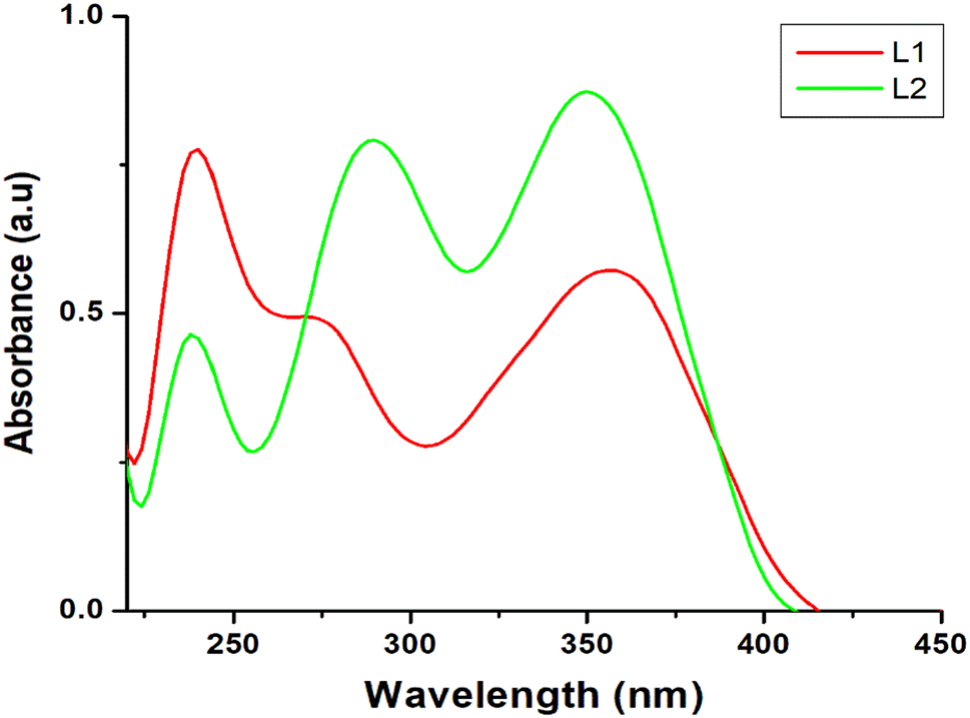
The electronic absorption spectra of the ligands obtained in dichloromethane.

The condensation of 2-aminophenol with the methoxybenzaldehydes produced the imine (C=N) functionality observed by a stretching band at 1587 and 1589 cm^−1^ on the IR spectra for **L1** and **L2** respectively (Figures S1 and S2). Additional bands observed around 3371, 2836, 1616, and 1250 cm^−1^ were assigned to ν(O–H), ν(C–H), ν(C=C) and ν(C–O), respectively ^3^. Similarly, ^1^H- and ^13^C-NMR spectra of the ligands (Figs. S3–S6) afforded the imine proton and carbon peaks at 9.21 and 153.1 ppm for **L1** as well as 8.65 and 162.5 ppm for **L2** (Figures S5 and S6), which were further established the formation of the Schiff base ^30^. The electronic absorption spectra of the ligands obtained in dichloromethane are shown in Fig. 2. Due to intra-ligand charge transfer observed between 238 and 274 nm, absorption bands were assigned to π → π* while the band around 350 nm was assigned to n → π* transition ^15^.

### Weight loss examination

#### Effect of inhibitors concentrations and temperature of test solution

The corrosion rate values of mild steel without and with varying concentrations of **L1** and **L2** Schiff base inhibitors at different temperatures were computed based on Eq. 2. The obtained results from measurement by weight loss techniques presented in Table 1 revealed that the inhibitors retard the rate of corrosion rate since a decrease in corrosion rate values was observed following an increasing concentration of the inhibitors from 0.0002 M to 0.001 M and invariably resulted in an increase in inhibition efficiency. A concentration of 0.001 M gave inhibition efficiency of 75% and 76% for **L1** and **L2** respectively at 303 K. The increase in inhibition efficiency obtained with increasing inhibitor concentration is related to surface coverage increase in the inhibitor molecules on the surface of the mild steel. This trend is in agreement with previous work^2,3^.

**Table 1:** Weight loss result of corrosion of mild steel in 0.1 mol L^−1^ HCl solution without and with varying concentrations of the inhibitors **L1** and **L2** at temperatures from 303 to 363 K.

Inhibitors		303 K			333 K			363 K		
	C_inh_ (ppm)	CR(mg/cm^2^h)	θ	η(%)	CR(g/cm^2^h)	θ	η (%)	CR(mg/cm^2^h)	θ	η (%)
**L1**	Blank	0.0033	–	–	0.0036	–	–	0.0039	–	–
	2 × 10^–4^	0.0017	0.50	50	0.0022	0.39	39	0.0028	0.29	29
	4 × 10^–4^	0.0014	0.58	58	0.0019	0.46	46	0.0025	0.36	36
	6 × 10^–4^	0.0014	0.58	58	0.0017	0.54	54	0.0022	0.43	43
	8 × 10^–4^	0.0011	0.67	67	0.0014	0.65	62	0.0019	0.50	50
	10 × 10^–4^	0.00083	0.75	75	0.0011	0.69	69	0.0017	0.56	56
	Blank	0.0033	–	–	0.0036	–	–	0.0039	–	–
**L2**	2 × 10^–4^	0.0022	0.33	33	0.0025	0.31	31	0.0028	0.28	28
	4 × 10^–4^	0.0019	0.42	42	0.0022	0.39	39	0.0025	0.36	36
	6 × 10^–4^	0.0014	0.58	58	0.0020	0.47	47	0.0022	0.44	44
	8 × 10^–4^	0.0011	0.67	67	0.0017	0.53	53	0.0019	0.51	51
	10 × 10^–4^	0.0008	0.76	76	0.0014	0.69	69	0.0017	0.56	56

**Figure 3:**
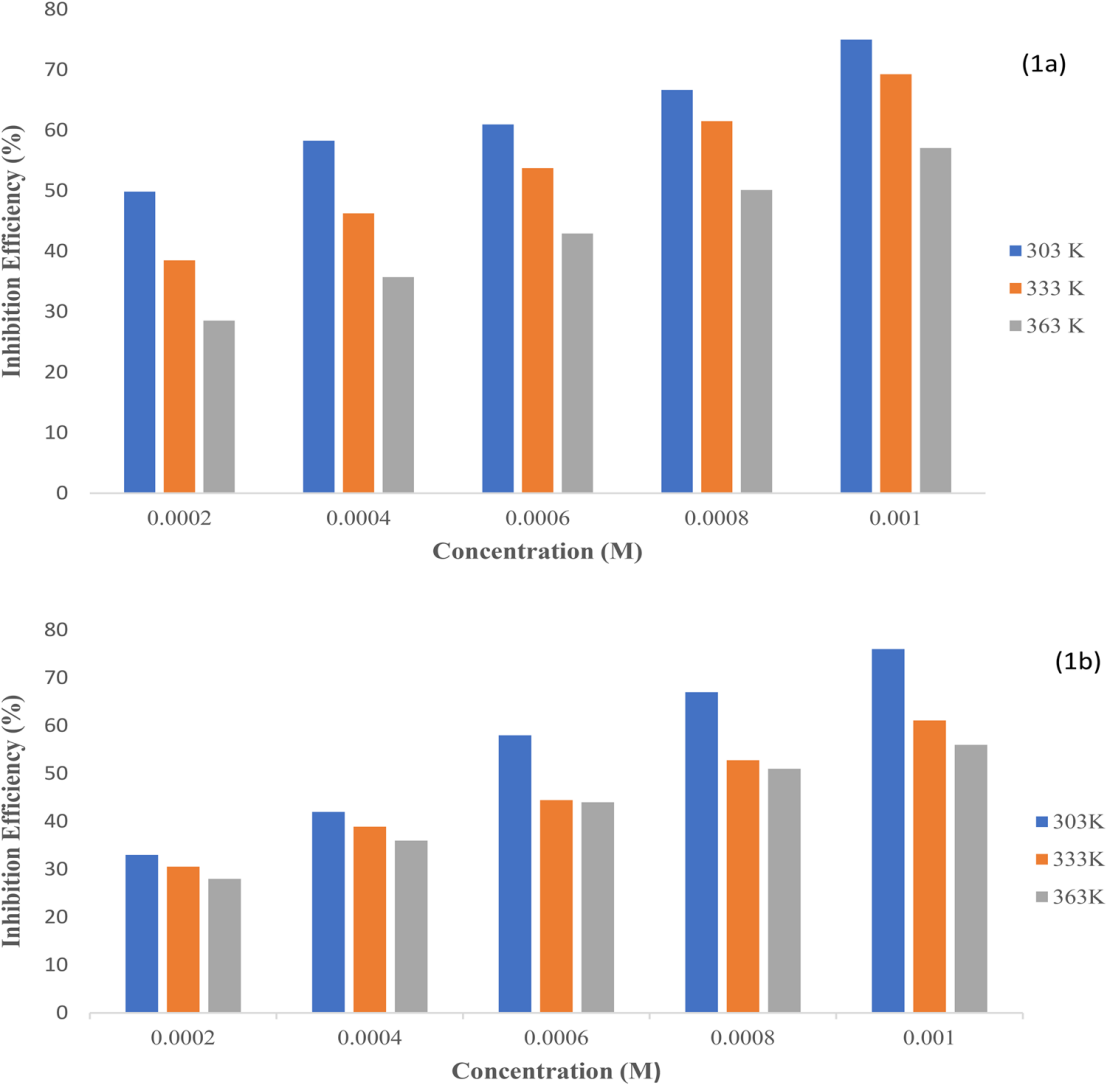
Effect of varying concentration of (**a**) **L1** and (**b**) **L2**, with varying temperature on inhibition efficiency.

Furthermore, it was observed that increasing solution temperature, led to a decline in inhibition efficiency for both **L1** and **L2** respectively (Fig. 3). This could be attributed to the desorption of molecules of the inhibitors from the surface of the mild steel which arises from kinetic energy increment of the inhibitor molecules which relapse the intermolecular force of interaction. An Increment in solution temperature could have also led to the decomposition of the molecule of the Schiff base inhibitors, hence, weakening surface coverage. The trend as observed in this study is in tandem with previous studies^3,31,32^. The Schiff base ligands showed comparable inhibitory efficiency at all temperatures (Table 1) with L2 slightly higher than L1 as a result of the electronic character of the methoxy substituent at the *para* and *ortho* positions respectively.

#### Adsorption isotherm and thermodynamic parameters

The inhibitor’s efficiency is attributed to the degree of metal surface coverage by the inhibitor, which is a function of the concentration of the inhibitor. This explains the nature of adsorbed inhibitors unto mild steel surface revealed from several studies^33,34^. In this case, the adsorption nature was elucidated by fitting data obtained from the weight loss experiment, unto the adsorption isotherm models of Langmuir and Freundlich. For both inhibitors of **L1** and **L2**, the Langmuir isotherm model (Eq. 19) gave a better correlation coefficient (R^2^) value closer to unity from the plot of C_inh_/θ vs. C_inh_ (Fig. S7) when compared to the Freundlich model. Hence, the Langmuir model better described the nature of the adsorption process.

19 $${\frac{C}{\theta }= \frac{1}{{K}_{ads}}+C}_{inh}$$

where C_inh_ is the concentration of the inhibitor (molL^−1^), K_ads_ is the adsorptive equilibrium constant (L mol^−1^) and θ is the surface coverage. The equilibrium constant values (K_ads_) were computed from the intercept of the plots of C_inh_/θ vs. C_inh_ (Figures S7). Table 2 revealed high K_ads_ values for both **L1** and **L2** which signifies the strong adhesiveness of the inhibitor molecules on the surface of the mild steel. The values of the standard free energy (ΔG_ads_) of the adsorption process were derived from the K_ads_ values according to Eq. (20).

20 $${\Delta G}_{{{\text{ads}}}}^{0} = \, - {\text{RTln}}({55}.{\text{5K}}_{{{\text{ads}}}} )$$

T is the absolute temperature (K), R = universal gas constant (8.314 J K^−1^ mol^−1^), 55.5 is the molar concentration of water in solution (mol/dm^3^). The computed ${\mathrm{\Delta G}}_{\mathrm{ads}}^{0}$ values (Table 2) of the adsorption process obtained were all negative, thereby, stipulates the occurrence of a spontaneous adsorption process.

**Table 2:** ${\mathrm{\Delta G}}_{\mathrm{ads}}^{0}$ and K_ads_ obtained from Langmuir Isotherm onto mild steel HCl (0.1 M) at varying concentrations, and temperatures of 303 K, 333 K and 363 K.

Inhibitor	Temperature (K)					
		K_ads_ (10^3^ L/mol)	${\mathrm{\Delta G}}_{\mathrm{ads}}^{0}$(kJ/mol)	Slope	R^2^	Intercept
**L1**	303 K	5.0	−31.57	1.1891	0.9799	0.0002
	333 K	2.5	−32.78	1.1427	0.970	0.0004
	363 K	2.0	−35.06	1.2896	0.9609	0.0005
**L2**	303 K	2.0	−29.266	0.8306	0.9388	0.0005
	333 K	2.0	−32.163	1.2254	0.9494	0.0005
	363 K	2.0	−35.060	1.3002	0.9793	0.0005

In general, if ${\mathrm{\Delta G}}_{\mathrm{ads}}^{0}$ values are about −20 kJ/mol or much lesser, it suggests that the process of adsorption occurs via the electrostatic interaction between the charged molecules and the charged metal surface. Hence, physisorption. Whereas, if values of ${\mathrm{\Delta G}}_{\mathrm{ads}}^{0}$ is approximately −40 kJ/mol or higher, it stipulates the sharing of charge or electron transfer between or from organic inhibitors to the surface of the metal resulting in the formation of a coordinate bond which is chemisorption^35^. However, values between − 20 and − 40 kJ mol^−1^of ${\mathrm{\Delta G}}_{\mathrm{ads}}^{0}$ stipulates the adsorption is a mixed type involving both physisorption and chemisorption^36^. Since the values of ${\mathrm{\Delta G}}_{\mathrm{ads}}^{0}$ computed in this study is the range of − 20 and − 40 kJ mol^−1^, hence, it connotes the adsorption was governed by both chemisorption and physisorption. Chemisorption tends to predominate since ΔG_ads_ values for both **L1** and **L2** are much closer to − 40 kJ mol^−1^. This explains that there exists transfer of electrons from the heteroatoms of the **L1** and **L2** to the surface of the metal leading to the formation of coordinate bonds.

#### Thermodynamic parameters of the corrosion reaction

The Arrhenius Eq. (21) was used to further gain an understanding of the kinetics involved with respect to the varying temperature effect on the corrosion rate of the mild steel in the acidic medium both in the presence and/or absence of the inhibitors used.

21 $$Log CR=Log A-\frac{{E}_{a}}{2.303R} .\frac{1}{T}$$

where CR is the corrosion rate, E_a_ is the apparent activation energy, and A is the Arrhenius pre-exponential factor, T is the absolute temperature (K). Figures S8, revealed the Arrhenius plot of the corrosion rate of the mild steel in 0.1 mol L^−1^ HCl, in the absence and presence of the varying concentrations of the inhibitors at temperatures of 303 K, 333 K, and 363 K for 3 h. The computed E_a_ values obtained from the slope of the plots (Fig. S8) are presented in Table 3 which shows that the least E_a_ values occur in blank solution and then increase with the addition of inhibitors. The activation energy increase is assigned to an increased energy barrier of the dissolution of the mild steel correlated to the adsorbed inhibitors barring the active sites on the high-energy metal surface. Moreover, a slightly higher E_a_ value was observed in the presence of **L2** at the highest concentration of 0.1 mM when compared to that of **L1** (Table 3). Furthermore, values of enthalpy of activation, (ΔH^∗^_corr_) and entropy of activation, (ΔS^∗^_corr_) for the process of corrosion were computed from the slope and intercept in the straight-line plot of $Log \left(\frac{CR}{T}\right)vs. \frac{1}{T}$(Fig. S9) of the Eyring transition state Eq. (22)^34^.

22 $$Log \left(\frac{CR}{T}\right)= -\frac{{\mathrm{\Delta H}}_{\mathrm{corr}}^{*}}{2.303RT} + \frac{{\mathrm{\Delta S}}_{\mathrm{corr}}^{*}}{2.303R} +Log \left(\frac{R}{{N}_{A}h}\right)$$

where N_A_ is the Avogadro number and h is the Planck constant. Other abbreviations have been defined in previous equations. The computed values of ΔH^∗^_corr_ and ΔS^∗^_corr_ are presented in Table 3. It was observed that the value of the activation enthalpy ΔH* is all positive, hence, revealing the endothermic nature of the mild steel dissolution reaction. Furthermore, it was observed that the least value of ΔH^∗^_corr_ occurs in the blank and the highest value of ΔH^∗^_corr_ occurs in the solution with the highest concentration of inhibitors. This could be best explained by that the minimum required energy needed by reactants to overcome energy barrier tend to increase with increasing inhibitors. The value of ΔH^∗^_corr_ at the highest concentration of 10 mM for **L2** > **L1**. In the case of the entropy of activation (ΔS_corr_^∗^), all the computed values were negative and quite large. This inclines that the activated complex is associative in nature in the determining step of the rate of reaction and the structure being more orderly, thereby resulting in entropy decline^3,25^. Also, Table 3 further shows that the computed value of ΔS_corr_ in the blank is more negative as compared to the ΔS_corr_ computed values of the varying concentration of the inhibited acid medium and this could be attributed to the presence of the adsorbed inhibitor species on the surface of the mild steel^9^.

**Table 3:** Activation energy (Apparent) (*E*_a_), A, ΔH^∗^, ΔS^∗^ of **L1** and **L2** at different concentrations derived from Arrhenius and Transition State Equations.

Inhibitor	Concentration(M)	Activation parameter			
		E_a_(kJ/mol)	A	ΔH^≠^ (J/mol)	ΔS^≠^ (J/mol K)
**L1**	Blank	2.37	0.126	23.44	−197.53
	2 × 10^–4^	7.77	0.238	32.42	−197.51
	4 × 10^–4^	8.96	0.270	34.50	−197.50
	6 × 10^–4^	8.12	0.225	33.73	−−197.51
	8 × 10^–4^	8.44	0.221	34.68	−197.507
	10 × 10^–4^	10.52	0.278	38.21	−197.499
**L2**	Blank	2.54	0.129	23.68	−197.539
	2 × 10^–4^	3.67	0.132	26.50	−197.539
	4 × 10^–4^	4.18	0.135	27.64	−197.539
	6 × 10^–4^	6.99	0.195	32.12	−197.537
	8 × 10^–4^	8.46	0.227	34.76	−197.537
	10 × 10^–4^	11.63	0.342	39.71	−197.535

### Surface analyses

**Figure 4:**
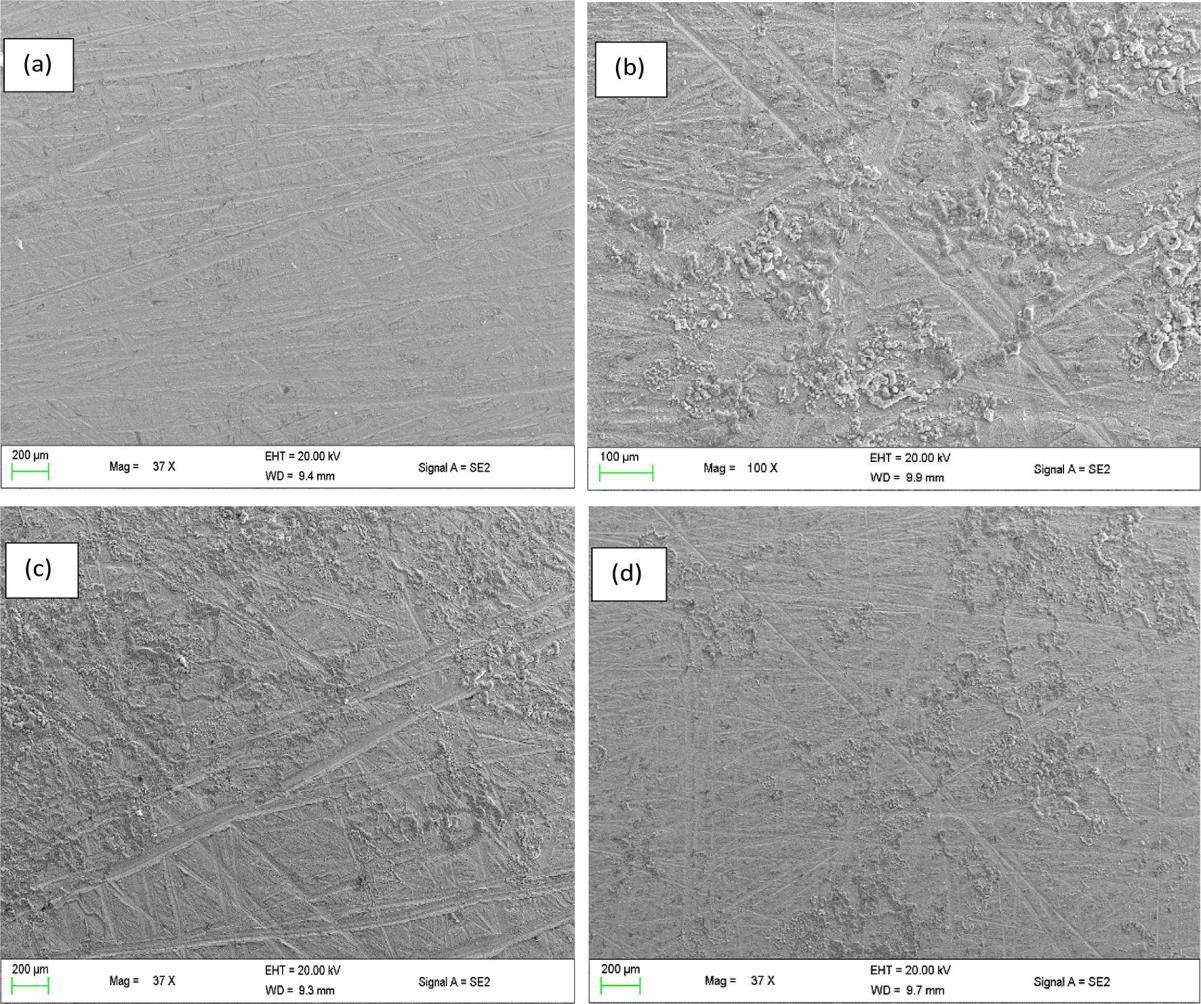
SEM micrographs of surface of mild steel (**a**) prior to immersion, (**b**) after 3 h immersion in 0.1 M HCl without inhibitors, (**c**) with 10 mM of **L1** and(d) **L2.**

The micrographs as shown from the scanning electron microscope (SEM) (Fig. 4) show the morphology and change in morphology on the surface of the mild steel prior to and after 3 h immersion in uninhibited and inhibited 0.1 M HCl solutions at 303 K. It was observed that the surface of the mild steel in the uninhibited acid solution was highly degraded with products of corrosion on its surface and this could be attributed to the increased dissolution rate from the direct attack on the surface. However, a mild degradation was observed on the surface of the mild steel immersed in the **L1** and **L2** inhibited acid solution, and this could be a result of mild steel surface coverage by both **L1**, and **L2** inhibitors, therefore, protecting it from corrosion by the HCl. This agrees theoretical studies as well as the weight loss results.

### EDX analysis

**Figure 5:**
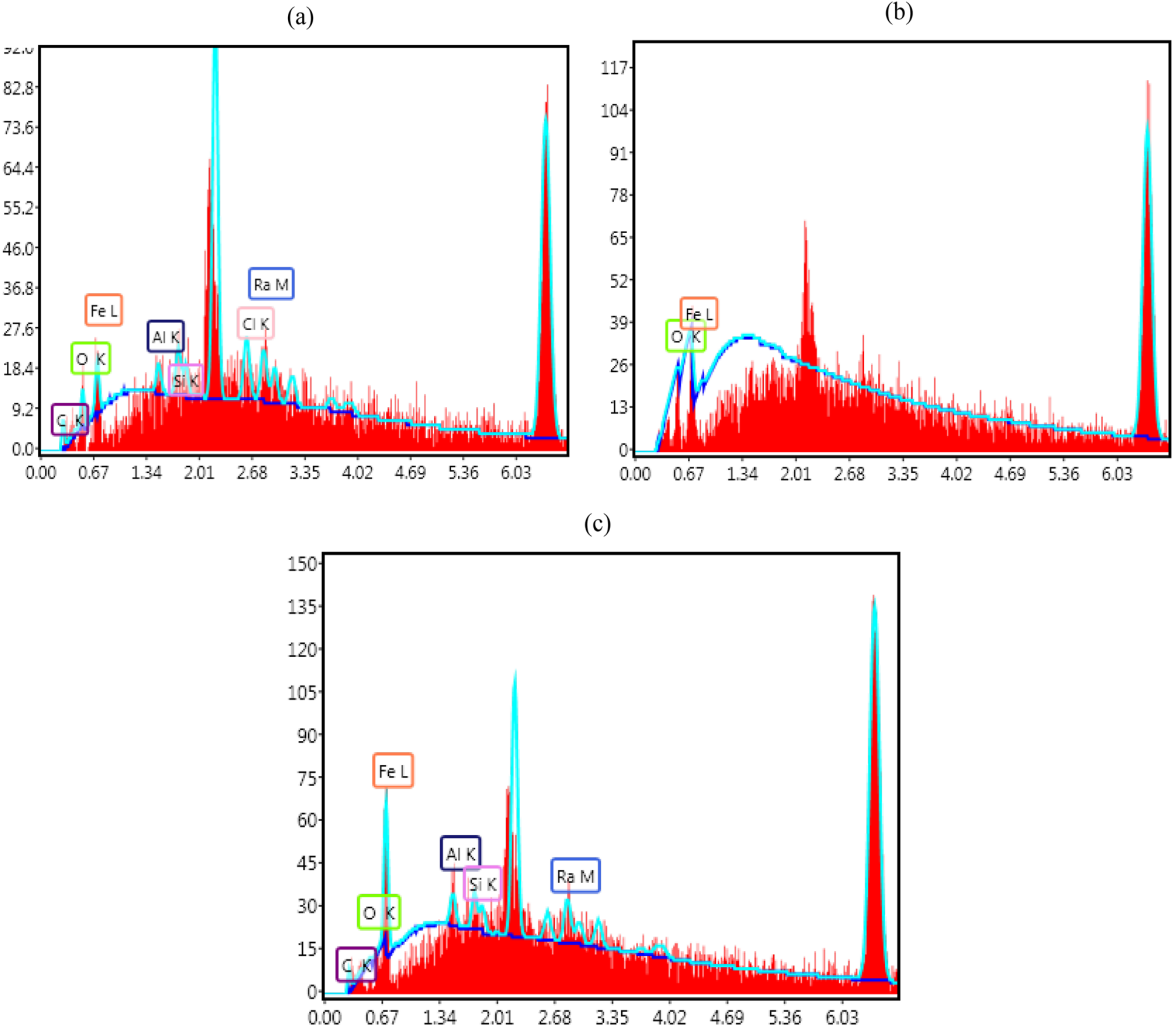
(**a**) Spectrum of mild steel in 0.1 M HCl (**b**) Spectrum of mild steel in 0.1 M HCl inhibited **L1** Schiff base, (**c**) Spectrum of mild steel in 0.1 M HCl inhibited **L2** Schiff base.

The EDX analysis was obtained to ascertain information with respect to the elemental constituent on the mild steel surface in 0.1 M HCl treated without and/or with inhibitors. The EDX spectrum of the mild steel coupons in 0.1 M HCl without and/or with 1 mM **L1**, and **L2** inhibitors respectively is revealed in Fig. 5. It can be seen from Fig. 5a, the existence of the peak of chlorine indicates the presence of this element on the mild steel surface that emanates from the hydrochloric acid solution. Furthermore, the displayed spectrum in Fig. 5b-c revealed the absence of chlorine which could be attributed to the application of the inhibitors on the mild steel surface acting as a coverage to decrease the corrosive attack of the acidic solution.

### Mechanism of inhibition

The mechanism involved in the adsorption of the inhibitor molecules on the metal surface from the weight loss experiment as observed from the computed thermodynamic parameters conforms to mixed adsorption of both physisorption and chemisorption with the predominance of chemisorption over physisorption. This posits the contribution of the charged protonated molecules of the **L1** and **L2** inhibitors onto the metal surface. The mechanism in chemisorption occurs via by formation of coordination bonds between the active sites on the metal (Fe) surface and the inhibitor molecules, and this coordination is made possible between the centres that donate and withdraw electrons.

### DFT analysis

Quantum mechanics method through conceptual DFT analysis has demonstrated suitability in the field of corrosion science for either acid or base medium used for metal corrosion inhibition study^37^. A number of studies^27,38^ have revealed the relationship between corrosion inhibition and the functional groups associated with the compounds under consideration. Analysis done with the B3LYP and M06-2X functional at 6-31 + G(d,p) basis set is presented.

### Optimized geometries and energies analysis

**Figure 6:**
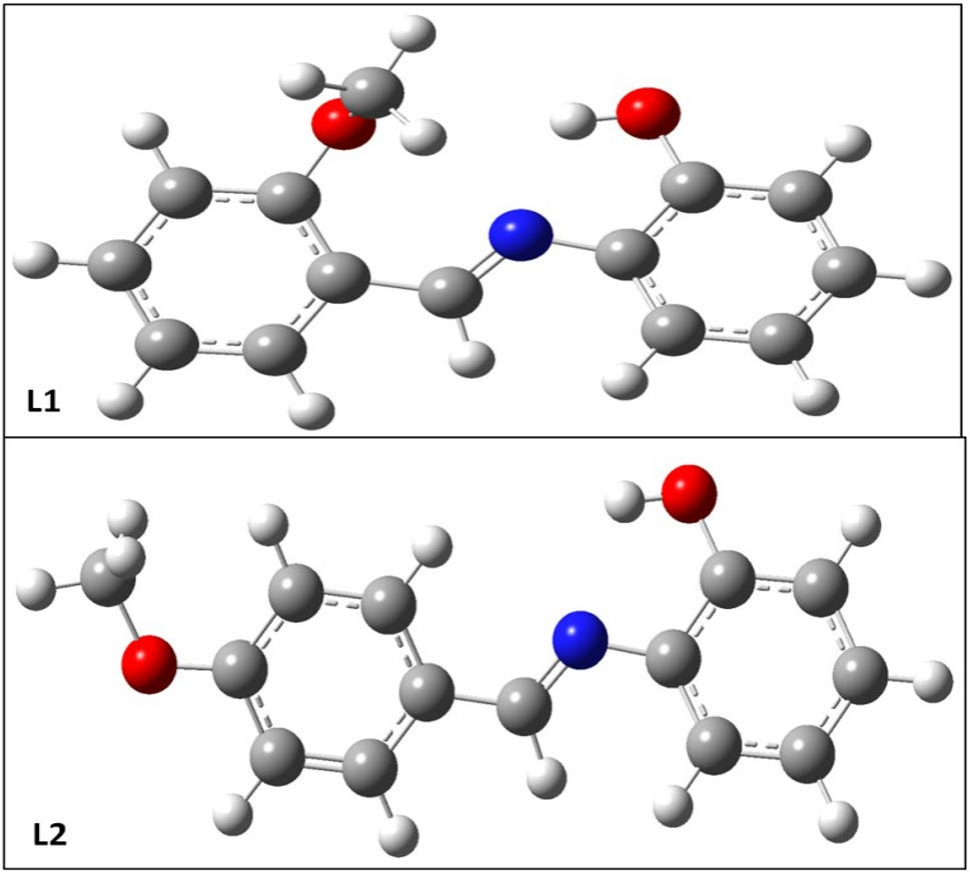
Optimized geometries of **L1** and **L2.**

Gaussian optimized structures of the Schiff bases are presented in Fig. 6.

The work of Lgaz et al.^38^ supports the idea that inhibitor adsorption on the iron surface can proceed on a donor and acceptor reactions basis which links the unoccupied surface atom orbit and n electrons of the aromatic compounds. Table 4 shows the energies of the LUMO and HOMO orbitals plus other conceptual DFT parameters. The higher HOMO energies obtained for both ligands in HCl solvent compared to gas-phase medium speed up the molecule’s binding to the metallic surface and thus interferes with the electron transfer mechanism over the adsorbed layer. The energy gap, ΔE measures the minimum energy required for exciting an electron in the inhibitor molecule^39^. This study reveals that the hybrid B3LYP functional performs better than M06-2X functional in measuring the energies of Schiff bases for corrosion inhibition because, the former gave much lower ΔE values of 3.86 eV and 3.81 eV for **L1** and **L2**, respectively as against 6.08 eV and 6.03 eV obtained with the later functional. Hence, the newly synthesized Schiff bases’ inhibitory efficiency is better measured and higher with hybrid B3LYP functional.

**Table 4:** Electronic parameters and reactivity descriptors.

	E_LUMO_ (eV)	E_HOMO_ (eV)	ΔE (eV)	A (eV)	I (eV)	η (eV)	δ (eV^−1^)	χ (eV)	C_p_ (eV)	ω (eV)	ΔN	μ (Debye)
L1, B3LYP (gas)	−2.16	−5.89	3.72	5.89	2.16	1.86	0.53	4.02	−4.02	4.35	0.79	3.49
L1, B3LYP (HCl)	−2.19	−6.06	3.86	6.06	2.19	1.93	0.51	4.12	−4.12	4.40	0.74	5.08
L1, M06-2X (gas)	−1.23	−7.17	5.94	7.17	1.23	2.97	0.33	4.20	−4.20	2.97	0.47	3.53
L1, M06-2X (HCl)	−1.28	−7.36	6.08	7.36	1.28	3.04	0.32	4.32	−4.32	3.07	0.43	5.00
L2, B3LYP (gas)	−1.99	−5.73	3.73	5.73	1.99	1.86	0.53	3.86	−3.86	3.99	0.83	3.57
L2, B3LYP (HCl)	−2.06	−5.88	3.81	5.88	2.06	1.90	0.52	3.97	−3.97	4.14	0.79	4.60
L2, M06-2X (gas)	−1.05	−7.02	5.96	7.02	1.05	2.98	0.33	4.03	−4.03	2.73	0.49	3.37
L2, M06-2X (HCl)	−1.14	−7.18	6.03	7.18	1.14	3.01	0.33	4.16	−4.16	2.87	0.46	4.16

Similarly, the chemical hardness is low in the hybrid B3LYP functional compared to M06-2X. This accounts for a relatively high softness and implies readily increased chemical reactivity as a corrosion inhibitor. The ΔN > 0 obtained for the ligand molecules reflects that they are very likely to donate electrons^40^. The viability and preference to use B3LYP functional over M06-2X for energies calculation are here confirmed since the former functional fraction of electron transferred as 0.74 and 0.79 (in HCl) for ligands **L1** and **L2** respectively as against 0.43 and 0.49 obtained using the later functional. The presence of aromatic rings and heteroatoms contributes to the corrosion inhibition efficacy of the molecules. By juxtaposing the two compounds using the energies analysis, **L2** is more effective as a corrosion inhibitor than the **L1** as demonstrated in the lower energy gap values and higher electron transferred fraction. In summary, the para-substituted ligand (**L2**) is relatively a stronger corrosion inhibitor of mild steel in HCl than its ortho-substituted (**L1**) derivative.

**Figure 7:**
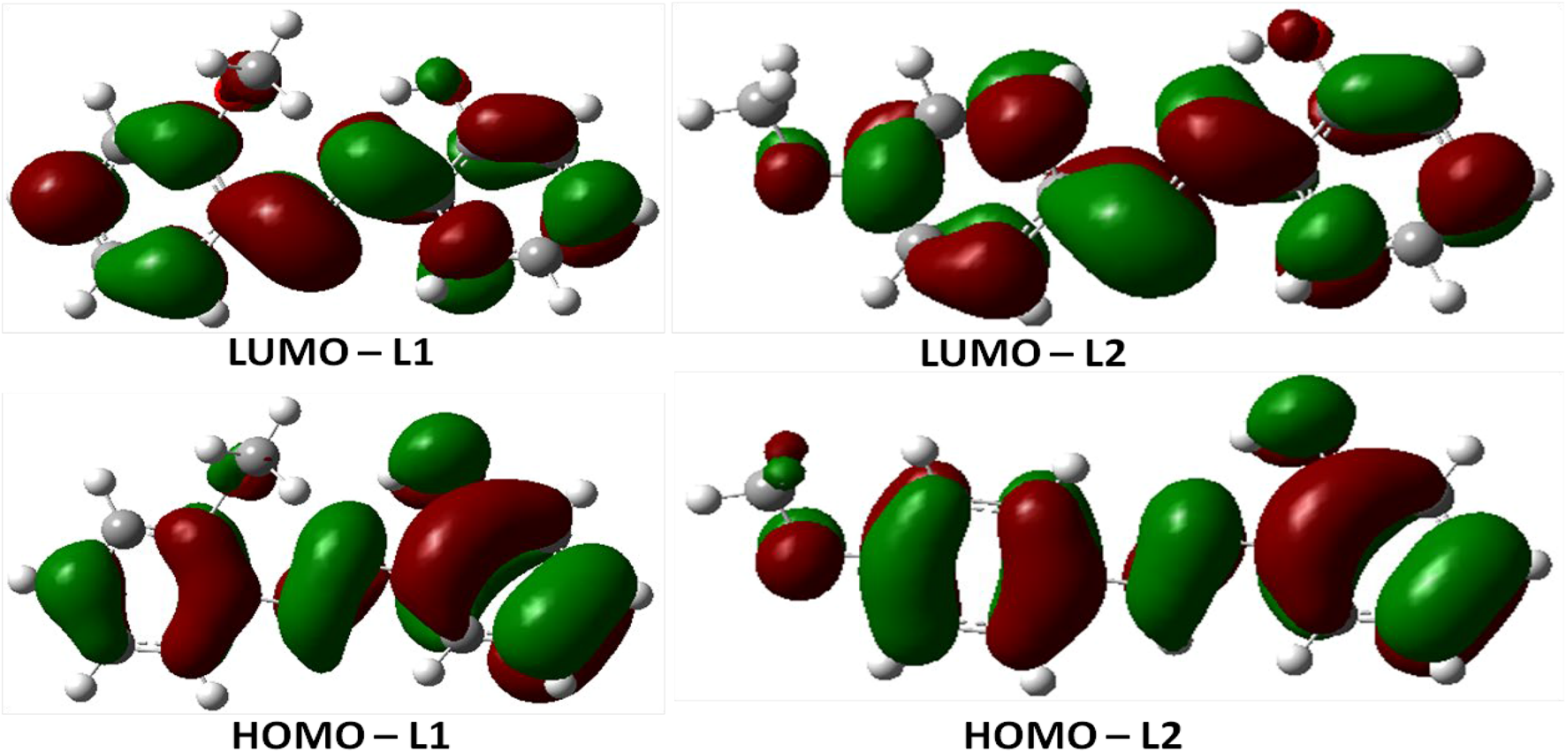
Frontier molecular orbitals of **L1** and **L2** calculated with B3LYP/6-31 + G(d,p).

The frontier molecular orbital maps of the Schiff bases obtained with B3LYP functional and 6-31 + G(d,p) basis set in HCl solvent are presented in Fig. 7. Maps from this model were chosen over the M06-2X model because the former gave a lower band gap which demonstrated a better measure of the electronic properties of the Schiff bases. Ortho-methoxyl substitution in **L1** partly affects the electron distribution in the phenyl group joined to C9 making the C9 atom electron-deficient and it is the preferred site for electrophilic attack. This is demonstrated in **L1** where the electron cloud (HOMO-**L1**) spread from the C6 atom towards the phenyl group attached to it. In the same vein, the electron cloud in L**2** spreads from the C6 atom towards the phenyl group attached to it, and the electron cloud in the phenyl group attached to C9 are evenly spread (HOMO-**L2**) and orbital maps facing each other. Thus, **L2** has a larger surface area with well-spread electrons for transfer and to be adsorbed onto mild steel and this supports the previous suggestion that **L2** relatively enhanced inhibition of mild steel corrosion than **L1** as observed in the weight loss study. This is also buttressed in the fraction of electrons transferred since ΔN is higher in **L2** than in **L1**.

### Calculation of Fukui indices and Mulliken atomic charges

The Mulliken population analysis provides means of estimating partial atomic charges in evaluating the adsorption centers of inhibition and it is employed in determining the diffusion load across the whole inhibitor molecule system ^27^. The Mulliken atomic charges and the respective Fukui function indices obtained are presented in appendices B3LYP /6-31 + g(d,p) calculation for **L1** shows that C6 (0.119) and C9 (−0.151) are the sites for nucleophilic and electrophilic attacks respectively in the gas-phase; while C15 (0.522) and C10 (−0.672) are the sites for nucleophilic and electrophilic attacks respectively in HCl solvent. On the other hand, using M06-2X/6-31 + G(d,p) for **L1** suggests that C6 (0.158, 0.227) and C9 (−0.159, −0.180) are the respective sites for nucleophilic and electrophilic attacks in gas-phase and gas-phase HCl solvent.

Using B3LYP/6-31 + G(d,p) for **L2** shows that C2 (0.104) and C10 (0.217) are the sites for nucleophilic attack both in gas-phase and in HCl solvent while C9 (−0.147, −0.205) is the preferred site for an electrophilic attack in both media. On the other hand, M06-2X/6-*31 + G(d,p) suggests that C2 (0.138) and C10 (0.385) are the sites for nucleophilic attack in gas-phase and HCl solvent respectively; while C11 (−0.205) and C9 (−0.445) are the sites for an electrophilic attack in gas-phase and HCl solvent. In summary, C6 and C9 are the respective preferred sites for a nucleophilic and electrophilic attack in **L1**; while C2 and C10 are the preferred sites for nucleophilic attack in gas-phase and HCl media while C9 is the preferred site for an electrophilic attack in **L2**.

### Molecular dynamics (MD) simulation

**Figure 8:**
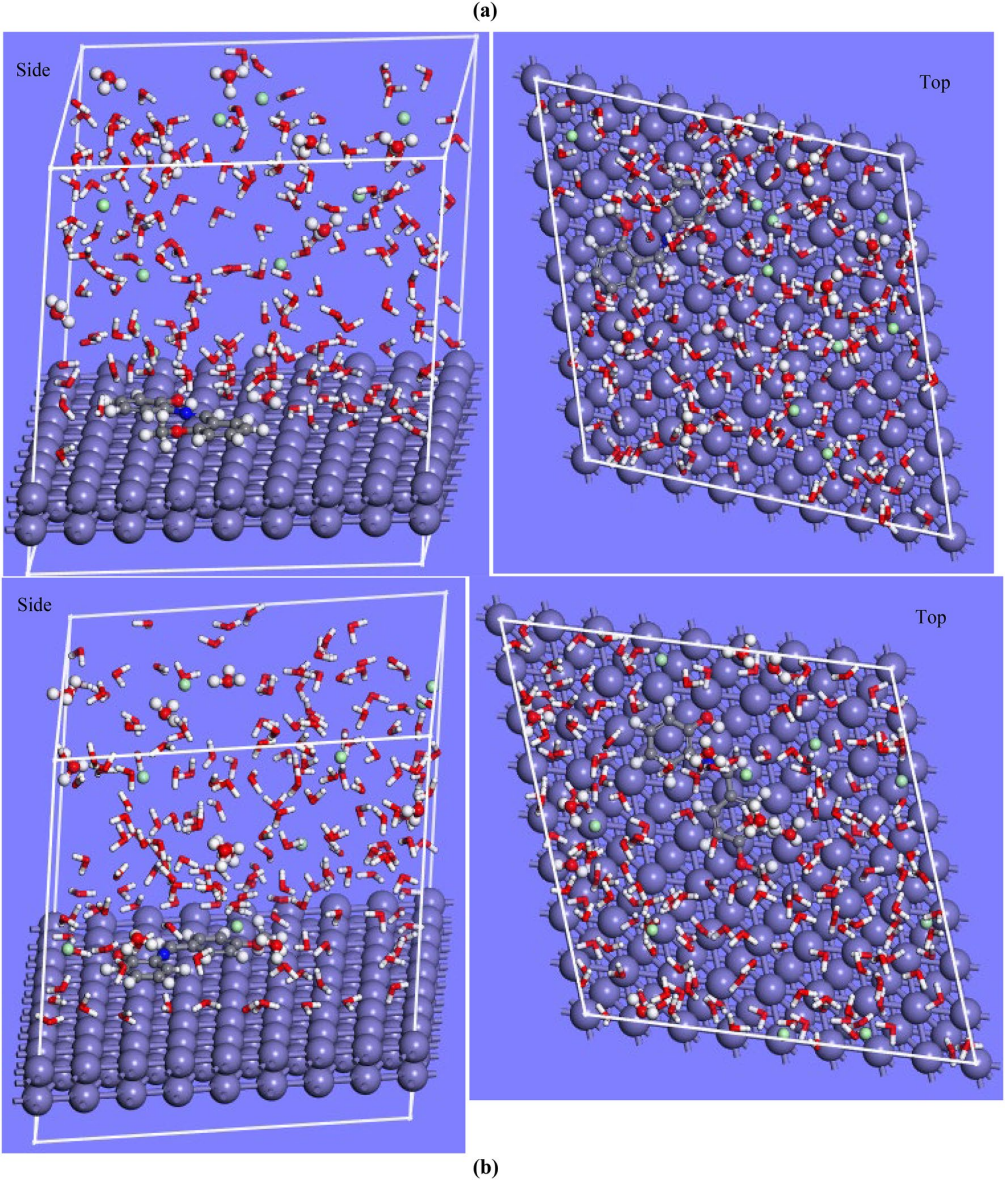
3D depiction of the Side and top views showing suitable conformation for adsorption of (**a**) **L1** and (**b**) **L2** on Fe (110) surface in aqueous solution.

Molecular dynamics simulation has been reported to be a recognized method introduced to corrosion studies^41^. This approach gives insight into the inhibitors’ interactions, orientation, and binding energies involving the inhibitors and metal surface^27,42^. In this work, MD simulations were adopted to complement the experimental results obtained and to study the mechanism of interactions between the inhibitors and the Fe (1 1 0) surface as well as in the presence of 200 water, H_3_O^+^ and Cl^−^. The surface configurations of the optimized inhibitors in the aqueous medium are presented in Fig. 8. MD simulations showed that **L1** and **L2** inhibitors adsorb with a parallel configuration (Fig. 8) firmly on the Fe (1 1 0) surface. This configuration resulted in the bond formation between the donor active site of **L1** and **L2** and the vacant orbitals of the positively charged Fe (1 1 0) surface^42^. The MD simulation revealed that notwithstanding the presence of the corrosive elements such as H_3_O^+^, Cl^−^ and the water molecules, a strong interaction exists between the surface of the metal and the heteroatoms (O, N, and the phenyl ring) of **L1** and **L2**. The interaction energy of compounds unto the Fe (1 1 0) surface was calculated and **L1** gave a value of −743.74 kJ/mol, while **L2** had a value of −746.84 kJ/mol. The large positive value obtained mirrors the ability of the compounds to adsorb on the surface of the metal surface. The calculated interaction energy obtained is higher compared to that reported for other compounds^27,43^. The MD results indicated that the interaction energy for **L1** and **L2** agreed with the inhibition efficiency obtained from the weight loss experiment. Based on the simulation, it can be inferred that the high interaction energy shows slightly higher adsorption capabilities of **L2** on the surface of the metal which also suggests better adsorption stability with higher inhibition efficiency^44^. The length of the bond between Fe and heteroatoms was measured to determine the mechanism of adsorption. For L1, Fe–O (2.89 Å), Fe–N (3.21 Å) and Fe–C (2.87 Å), while for L2, Fe–O (3.09 Å), Fe–N (3.33 Å) and Fe–C (2.73 Å). Bond lengths between 1 and 3 Å indicate chemisorption while ˃3.5 Å suggests a physisorption mechanism^27^. Analysis showed that all the bond lengths of studied molecules with Fe surfaces are less than 3.5 Å indicating a chemisorption mechanism.

### Inhibition mechanism

The corrosion inhibition mechanism of inhibitors depends largely on the adsorption of molecules on the metal surface which can be traced to factors such as the chemical structure of molecules under study^2,45^. The corrosion inhibition mechanism of compounds has been studied experimentally and via a theoretical approach. In this section, the mechanism of adsorption is discussed putting into consideration other components in the solution and the type of adsorption that occurs between Fe and **L1** and **L2. L1** and **L2** being Schiff base compounds could exist in the protonated form in 1.0 M Hydrochloric acid solution as seen in the following equilibrium:

23 $$\mathrm{L}\left(\mathrm{y}\right)+ {H}^{+}\leftrightarrow {L\left(y\right)H}^{+}$$

where y = 1, 2.

**Figure 9:**
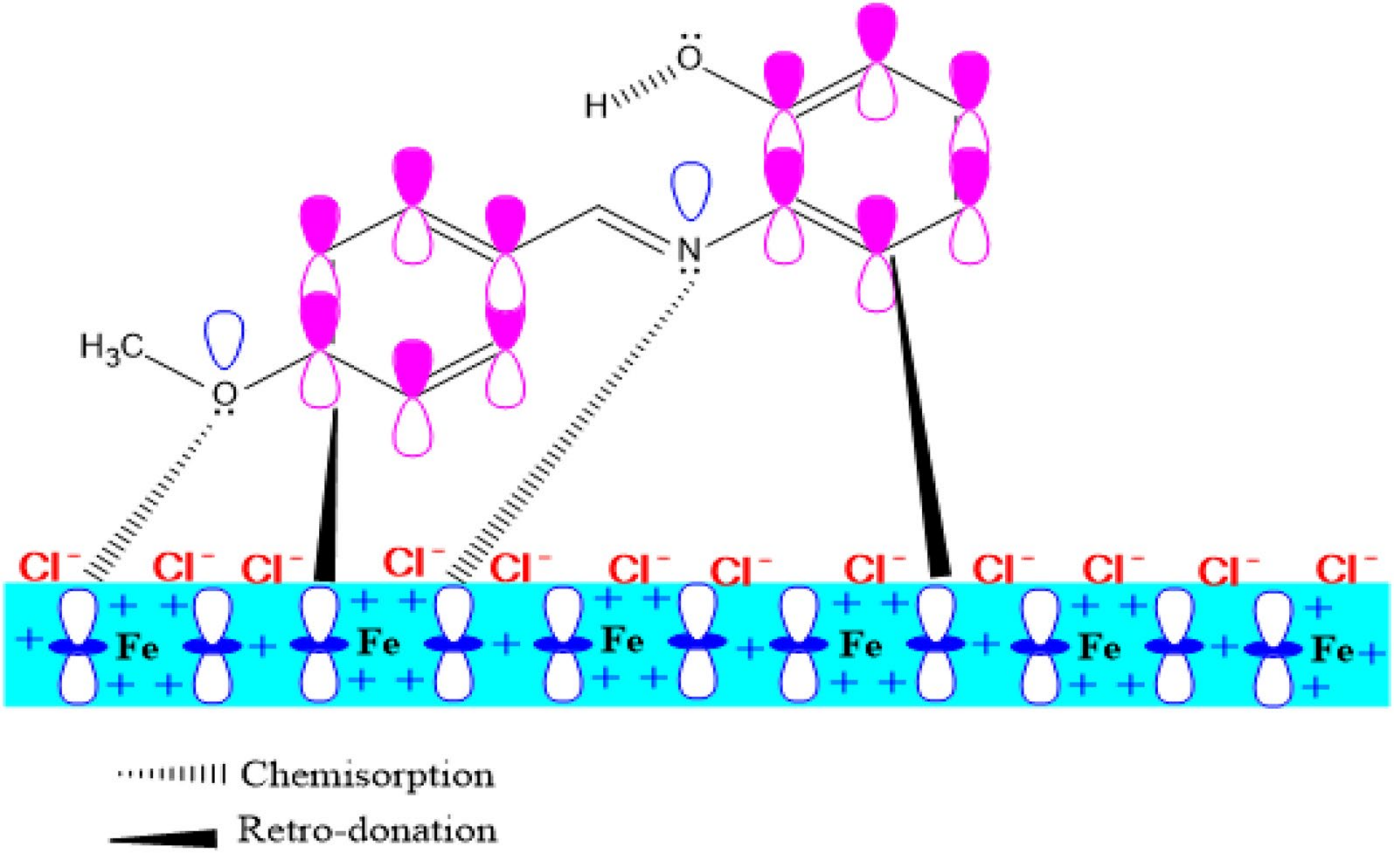
Schematic representation of the adsorption mechanism of inhibitors on the Fe (1 1 0) surface in 0.1 M HCl.

The resulting protonated form as shown in Eq. (23), shows that the adsorption of inhibitors on the metal surface promotes chemisorption. This is due to the electrostatic attraction that exists between the hydrated negative charges of Cl^−^ on the surface of Fe (1 1 0) and the positive charges from the ligands^2,45^. Hydrogen gas is released as the protonated form of the Schiff base competes with the aqueous H^+^, and the heteroatoms of the ligands return to their neutral form, which then transfers the unshared electron pair into the vacant orbitals of the Fe surface^2^. The series of transfers of electrons to the metal surface causes the accumulation of electrons in the metal. This results in a back the sequential transfer of electrons from the dd-orbitals of metal atoms to the unoccupied orbitals of the Schiff base leading to a phenomenon called chemisorption and retro-donation^42,46,47^. The isotherm study revealed that Langmuir Isotherm best fits the corrosion inhibition process and the values of ${\mathrm{\Delta G}}_{\mathrm{ads}}^{0}$ further suggest that the mechanism of corrosion inhibition by **L1** and **L2** is by chemisorption. This results from the transfer of lone pair of electrons from the Schiff base formed and the pi electrons of the phenyl ring to the metal surfaces forming coordinate bonds. Figure 9 shows a pictorial explanation of the mechanism of corrosion inhibition.

## Conclusion

Schiff base ligands (E)-2-((2-methoxybenzylidene)amino)phenol and (E)-2-((4-methoxybenzylidene)amino)phenol were synthesized and characterized using NMR, IR and UV/Visible spectroscopy. The corrosion inhibition potential of these synthesized compounds was investigated by weight loss measurement. Computational approaches such as molecular dynamics and the DFT approach were applied to further explain the mechanism of inhibition. DFT calculations were also applied to elucidate their chemical reactivity and inherent properties. In this study, the inhibition efficiency increased with increasing inhibitor concentration, and decreased with an increased temperature. Langmuir Isotherm best fits the experimental data obtained for **L1** and **L2**. DFT calculations showed that the presence of aromatic rings and heteroatoms contribute to the corrosion inhibition efficacy of the synthesized compounds. The interaction energies obtained for **L1** and **L2** from molecular dynamics studies agree with the DFT calculated parameters and the inhibition efficiencies obtained from the weight loss experiment. Chemisorption mechanism was inferred based on the value ΔG_ads_ and the close bond distance obtained between the heteroatoms of the ligands and the surface of the metal.

## Supplementary Information

Supplementary Information.

## Data Availability

The data that support the findings of this study are available from Collins U. Ibeji, but restrictions apply to the availability of these data, which were used under license for the current study, and so are not publicly available. Data are however available from the authors upon reasonable request and with permission of Collins U. Ibeji.
