# Supplementary material for: Synthesis, experimental and computational studies on the anti-corrosion performance of substituted Schiff bases of 2-methoxybenzaldehyde for mild steel in HCl medium
Source: Sci Rep. 2023 Feb 24;13:3265. doi: 10.1038/s41598-023-30396-3 (PMC9958021; doi:10.1038/s41598-023-30396-3)
Supplement: Supplementary file 1 — Supplementary Information. [file 41598_2023_30396_MOESM1_ESM.docx]

**Supplementary Material**

**Synthesis, Experimental and Computational studies on the Anti-Corrosion Performance of Substituted Schiff Bases of 2-Methoxybenzaldehyde for Mild Steel in HCl Medium**

Collins U. Ibeji*^1,2^, Damilola C. Akintayo^3^*, Henry O. Oluwasola^1^, Eric O. Akintemi^3^, Onyinye G. Onwukwe^1^, Onyeka M. Eziomume^1^

^1^Department of Pure and Industrial Chemistry, Faculty of Physical Sciences, University of Nigeria, Nsukka 410001, Enugu State, Nigeria.

^2^Catalysis and Peptide Research Unit, School of Health Sciences, University of KwaZulu-Natal, Westville Campus, Durban 4041, South Africa.

^3^School of Chemistry and Physics, University of KwaZulu-Natal, P.M.B. X54001, Durban, 4000, South Africa.

^4^Industrial Chemistry Unit, Department of Computer and Physical Sciences, Wesley University, P. M. B. 507, Ondo, Nigeria.

***Corresponding authors:** ugochukwu.ibeji@unn.edu.ng (C. U. Ibeji). ORCID: 0000-0003-4762-2256; damilolaakintayo141@gmail.com (D. C. Akintayo). ORCID: 0000-0003-1742-3571

**Figure S1:** FTIR of (E)-2-((2-methoxybenzylidene)amino)phenol **(L1)**

**Figure S2:** (E)-2-((4-methoxybenzylidene)amino)phenol (**L2)**


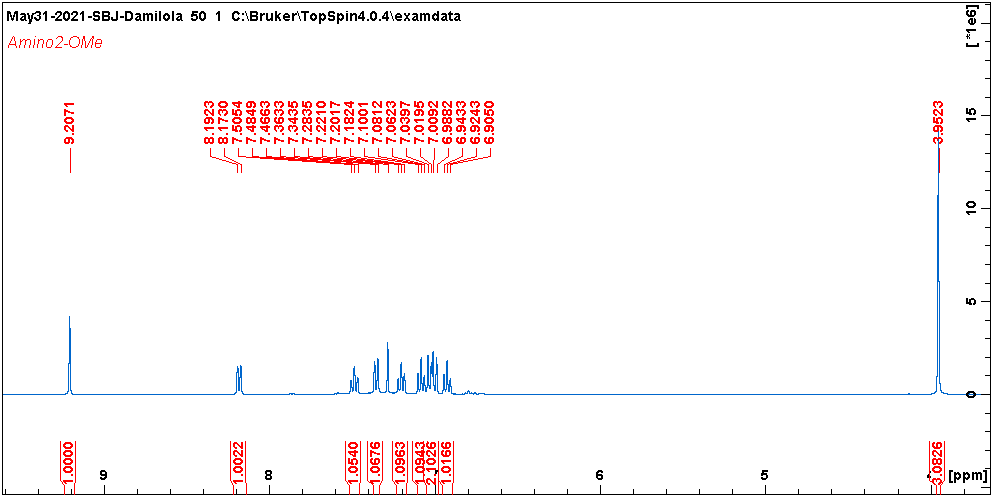


**Figure S3:** ^1^H NMR(E)-2-((2-methoxybenzylidene)amino)phenol (**L1)**


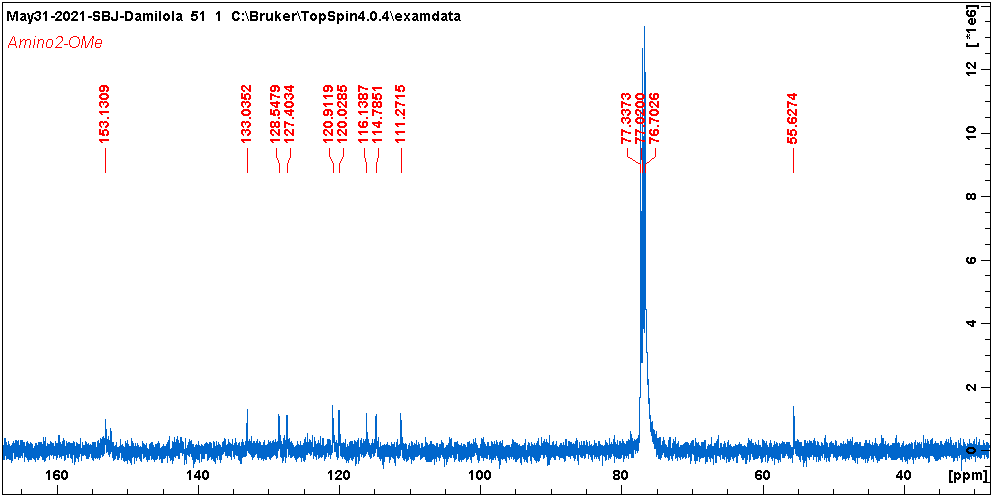


**Figure S4:** ^13^C NMR(E)-2-((2-methoxybenzylidene)amino)phenol (**L2)**


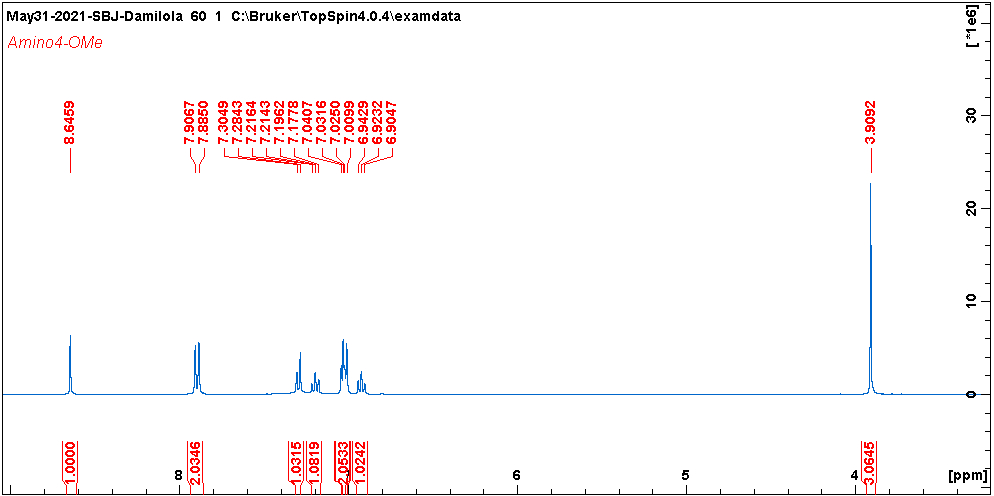


**Figure S5:** ^1^H NMR(E)-2-((4-methoxybenzylidene)amino)phenol (**L2)**


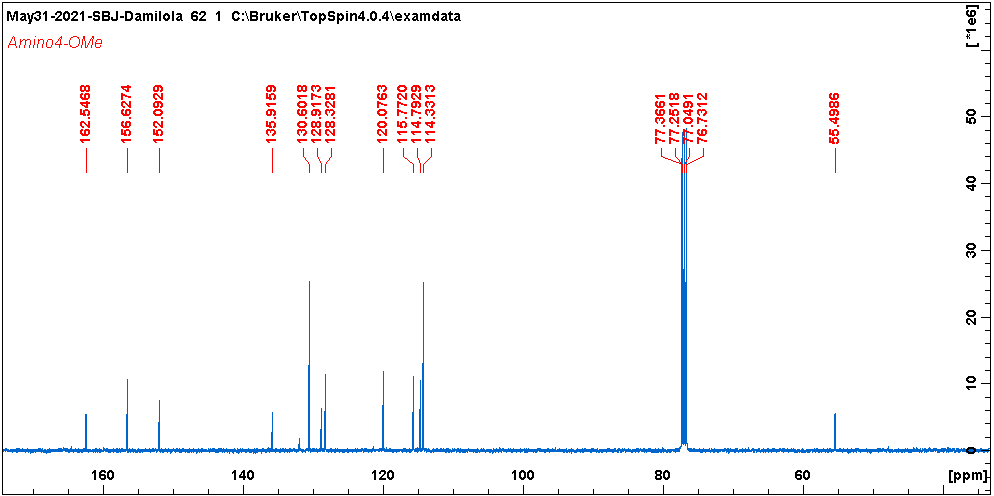


**Figure S6:** ^13^C NMR(E)-2-((4-methoxybenzylidene)amino)phenol (**L2)**

**Figure S7:** Langmuir adsorption isotherm plots for mild steel in 0.1 M HCl containing various concentrations of (a) L1 and (b) L2 inhibitor at temperatures of 303 K, 333 K, and 363 K for 3 h

**Figure S8:** Arrhenius plot of mild steel in 0.1M HCl medium in blank or varying concentration of (a) L1 and (b) L2

**Figure S9:** Transition state plot of log CR/T vs. 1/T for mild steel corrosion in 0.1 mol L^−1^ HCl in the absence and presence of varying concentration of (a) L1 and (b) L2
